# The effectiveness of Salvianolate injection for in-stent restenosis after percutaneous coronary intervention: A protocol for systematic review and meta-analysis

**DOI:** 10.1097/MD.0000000000029153

**Published:** 2022-04-22

**Authors:** Miao Zhang, Yue Yuan, Ying Gao, Ruozhu Lu, Yue Deng

**Affiliations:** a Doctor of Medicine, School of Traditional Chinese Medicine, Changchun University of Chinese Medicine, Changchun, China; b Doctor of Medicine, School of Basic Medicine, Changchun University of Chinese Medicine, Changchun, China; c Affiliated Hospital, Changchun University of Chinese Medicine, Changchun, China.

**Keywords:** coronary heart disease, in-stent restenosis, percutaneous coronary intervention, Salvianolate injection

## Abstract

**Background:**

In-stent restenosis (ISR) caused by vascular remodeling after percutaneous coronary intervention limits the long-term efficacy of this method. Salvianolate injection is now widely used in the clinical treatment of ISR. However, there is no systematic review or meta-analysis to evaluate the effects of Salvianolate injection on ISR.

**Methods:**

We will search articles in 8 electronic databases, including the Cochrane Central Register of Controlled Trials, PubMed, Embase, the Web of Science, China National Knowledge Infrastructure, the Chinese Biomedical Literature Database, Wanfang Database, and the Chinese Scientific Journal Database for randomized controlled trials of ISR treated by Salvianolate injection from their inception to February 27, 2022. The primary outcome measure will be the restenosis rate. The data meeting the inclusion criteria were analyzed by RevMan V.5.4 software. Two authors evaluated the study using the Cochrane collaborative risk bias tool. We will use a scoring method to assess the overall evidence supporting the main results.

**Results:**

This study will analyze the clinical effectiveness of Salvianolate injection in the treatment of ISR.

**Conclusion:**

The findings of this systematic review will provide evidence to evaluate the effectiveness of Salvianolate injection for the treatment of ISR.

**INPLASY registration number:**

INPLASY202220117.

## 1 Introduction

Percutaneous coronary intervention (PCI) is an essential method for the treatment of coronary heart disease (CHD).^[1]^ However, in-stent restenosis (ISR) caused by vascular remodeling after PCI limits the long-term efficacy of this method.^[2]^ High-resolution intracoronary imaging has shown that in the case of advanced stent failure after surgery, new atherosclerosis in the stent segment is the final common pathway of ISR events.^[3]^ Neointimal hyperplasia is considered to be the leading cause of the development of new atherosclerosis leading to ISR after PCI.

Drug-eluting stents (DES) and long-term anti-platelet aggregation drugs are often used clinically to prevent and treat ISR.^[4]^ However, the therapeutic effect is not satisfactory, while long-term use of anti-platelet drugs may cause gastrointestinal adverse reactions.^[5]^ Salvianolate injection could treat CHD in clinical practice.^[6]^ Salvianolate is mainly derived from the Chinese herb Salvia miltiorrhiza. Animal experiments have shown that salvia polyphenols can protect blood vessels by inhibiting the expression of NLRP3 inflammasome.^[7]^ Salvianolate injection is widely used in the clinic and has the advantages of low irritation to the gastrointestinal tract and high safety of drug administration. However, the effective clinical rate of Salvianolate injection for ISR still remains unclear. It still needs further exploration. This study aims to support the effective clinical rate of Salvianolate injection in ISR treatment, which will help clinicians widely use it in clinical treatment.

### 1.1 Design and registration information for the systematic review

This systematic review protocol has been registered with the International Platform of Registered Systematic Review and Meta-Analysis Protocols (INPLASY) and has been assigned the registration number INPLASY202220117. The study aims to evaluate the effectiveness of Salvianolate injection for use in the treatment of ISR.

### 1.2 Types of studies

We will collect published randomized controlled trials (RCTs) to evaluate the clinical efficacy, functional improvement results, quality of life, and adverse reactions of Salvianolate injection on ISR for systematic review and meta-analysis. All eligible tests will be included regardless of language and publication type. Review articles, case series, cohort studies, retrospective studies, and animal experiments that do not meet the requirements will be excluded.

### 1.3 Types of patients

All patients must meet the diagnostic criteria for adult CHD established by the American College of Cardiology/American Heart Association,^[8]^ all patients must have undergone PCI and have a diagnosis of ISR after PCI. There will be no limits placed on basic patient characteristics (including region, race, and sex).

### 1.4 Types of interventions

In the intervention group patients received Salvianolate injection or combined with another intervention.

In the control group, patients received another intervention or no treatment. Comparisons to be examined included the following.

- Salvianolate injection versus no Salvianolate injection.
- Salvianolate injection versus another intervention.
- Salvianolate injection combined with another intervention versus Salvianolate injection.

### 1.5 Types of outcome measures

#### 1.5.1 Primary outcomes

The primary outcome measure will be the restenosis rate.

#### 1.5.2 Secondary outcomes

The secondary outcomes will include the angina recurrence rate and major adverse cardiac events (MACEs).

### 1.6 Search strategy

We will search the following databases from their inception to February 27, 2022: The Cochrane Library, Embase, PubMed, Web of Science, China National Knowledge Infrastructure Database (CNKI), Wan-fang Database (wan-fang), Chinese Scientific Journals Database (VIP), and Chinese Biomedicine Database (CBM). The publishing language will be restricted to Chinese and English.

The following subject words were used: (“percutaneous coronary intervention” or “percutaneous coronary artery intervention” or “PCI” or “restenosis” or “in-stent restenosis” or “ISR”) and (“Salvianolate injection” or “depside salt from salvia miltiorrhiza” or “Dan shen duofen suan yan” or “Danshen polyphenolate injection”) and (“randomized controlled trial” or “randomized” or “trial”). The reference list of all selected articles will be independently screened to identify additional studies left out in the initial search. Table 1 provides details of the PubMed database search strategy. Other databases use the same search strategy. The reference list of relevant research articles was reviewed for additional article search.

**Table 1:** The search strategy for PubMed database.

Number	Search terms
#1	percutaneous coronary intervention
#2	percutaneous coronary artery intervention
#3	PCI
#4	in-stent restenosis
#5	restenosis
#6	ISR
#7	OR #1-#6
#8	Salvianolate injection
#9	depside salt from salvia miltiorrhiza
#10	Danshen duofen suan yan
#11	Danshen polyphenolate injection
#12	OR #8-#11
#13	randomized controlled trial
#14	randomized
#15	trial
#16	OR#18-#22
#17	#7 and #12 and #16

### 1.7 Study selection and data extraction

**Figure 1:**
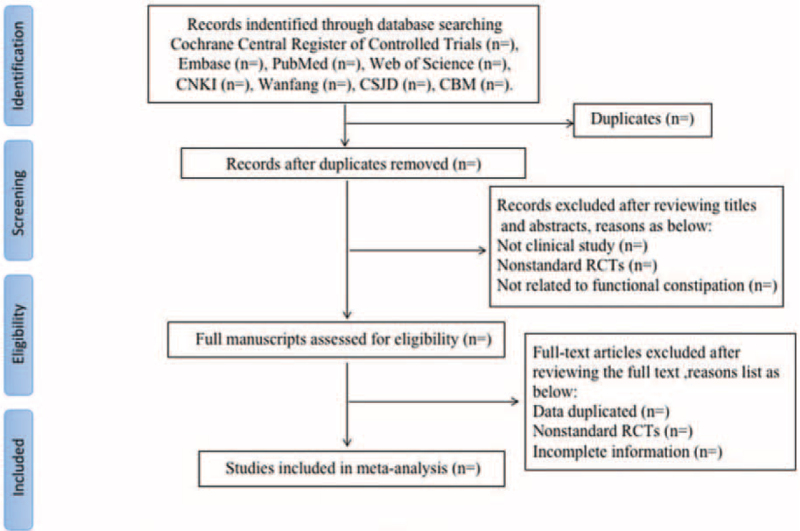
Flow chart of the search process.

Two review authors (ZM and GY) will independently evaluate the titles and abstracts of all citations found from the above search strategy. We will obtain the full text of all potentially relevant articles to assess further assess eligibility based on the inclusion/exclusion criteria. Disagreements will be resolved by consensus or mediated by a third author (YY). The final selection process will follow the PRISMA guidelines,^[9]^ as shown in Figure 1. Inclusion criteria were: studies that were RCTs comparing Salvianolate injection with a control group; patients in the studies had coronary atherosclerosis disease diagnosed by coronary arteriography and had successfully undergone PCI; patients were randomized to either the Salvianolate injection group or a control group; the primary outcome was angiographic restenosis, defined as stenosis >50% of the diameter, and secondary outcomes were MACEs, consisting of Myocardial infarction, repeated angioplasty, coronary artery bypass surgery, and death; and patients were followed for at least 6 months.

Exclusion criteria were: studies comparing the same medicines with different doses of time, frequency, or duration; conference abstracts without full published articles; duplicate data or data that cannot be extracted; and participants were diagnosed with ISR with other cardiovascular system diseases.

Finally, 2 authors (ZM and GY) will independently extract data using data extraction tables as recommended by the Cochrane Handbook for Systematic Reviews of Interventions. The following data will be extracted: study characteristics such as author, year of publication, country in which the study was conducted, study period, original inclusion criteria, and a total number of people included in the study; population characteristics such as mean age and time from diagnosis; intervention characteristics such as type, duration, and frequency; and outcomes such as the rate of clinically effective, angina recurrence, and MACEs. Any disagreements will be resolved by discussion until consensus is reached or by consulting a third researcher (YY). We will also contact the original authors of papers via email or telephone if possible.

### 1.8 Missing data management

We will contact the study authors via email or telephone to obtain missing data and additional information if possible. Otherwise, we will analyze the available information and conduct sensitivity analysis to explore the potential impact of insufficient information on the results of the meta-analysis.

### 1.9 Risk of bias assessment

Two review authors (ZM and GY) will independently evaluate each included study and will follow the domain-based evaluation as developed by the Cochrane Handbook for Systematic Reviews of Interventions. They will assess the following domains: selection bias (random sequence generation and allocation concealment); performance bias (blinding of participants and personnel); detection bias (blinding of outcome assessment); attrition bias (incomplete outcome data); reporting bias (selective reporting); and other bias (such as pre-sample size estimation, early stop of trial). Each domain will be divided into 3 categories: low risk of bias, unclear bias, and high risk of bias. Any discrepancies will be resolved by reviewing the original article and discussed with the third author (YY) to reach a consensus.

### 1.10 Data synthesis and analysis

We will analyze the data with RevMan V.5.4 software provided by The Cochrane Collaboration. A meta-analysis using random or fixed-effects models will be conducted to summarize the data. Continuous data will be pooled and presented as mean differences or standardized mean differences with their 95% confidence interval. Dichotomous data will be pooled and expressed as a risk ratio with their 95% confidence interval. We will interpret it using the following criteria: I^2^ values of 25% are considered low levels of heterogeneity, 50% indicated moderate levels, and 75% indicated high levels. Since low or moderate heterogeneity suggests little variability among these studies, the data will be analyzed in a fixed-effects model. When significant heterogeneity occurs among the studies (*P* < .05, I^2^ ≥ 50%), a random effect model will be performed to analyze the data.

### 1.11 Assessment of reporting biases

Reporting bias will be assessed by Egger regression asymmetry test.^[10]^ A *P* value < .05 in Egger test is considered statistically significant. STATA V.16.0.b software (Solvusoft Corporation, USA) will be used to perform the Egger test.

### 1.12 Subgroup analysis

We plan to carry out the following subgroup analyses to explore possible sources of heterogeneity: type of PCI. If the data are insufficient, the qualitative synthesis will be conducted instead of the quantitative synthesis.

### 1.13 Sensitivity analysis

Sensitivity analysis will be performed to examine the robustness of the results by eliminating low-quality trials.

### 1.14 Grading the quality of evidence

The quality of evidence regarding patient outcomes will be used assessed by the Grading of Recommendations Assessment, Development, and Evaluation methodology.^[11]^ It will be used to summarize the limitations in design, consistency, directness, precision, and publication bias. The quality of each piece of evidence will be divided into 4 levels: high, medium, low, and very low. Disagreements will be resolved by consensus.

## 2 Discussions

CHD is an important disease that seriously threatens human life and health. In recent years, the morbidity and mortality of CHD have increased each year, and CHD has become the number 1 factor affecting human health.^[12]^ PCI is a common treatment for CHD. PCI mainly includes percutaneous coronary angioplasty, coronary artery stenting, and rotational atherectomy. Percutaneous coronary angioplasty alone has a high incidence of acute occlusion and restenosis of coronary arteries. Therefore, coronary artery stenting is now mostly used. With the continuous progress of medicine, DES have now gradually replaced bare-metal stents in clinical practice. DES can further reduce ISR incidence compared with bare-metal stents. Although PCI technology is constantly updated, 15% to 20% of patients suffer restenosis within 3 to 6 months after undergoing PCI. Therefore, most patients use long-term use of anti-platelet or anticoagulant drugs to prevent the development of ISR. These include aspirin, clopidogrel, Tegretol, and low molecular heparin sodium. However, these drugs may carry the risk of gastrointestinal adverse effects and bleeding.

Chinese herbal medicine is a valuable resource to identify new small-molecule drugs against human disease. In recent years, traditional Chinese medicine (TCM) practitioners and clinicians have also integrated TCM with modern medicine, resulting in substantial improvement to dealing with cardiovascular and cerebrovascular diseases, with significantly reduced mortality and a much better quality of life. Danshen effective drugs to treat cardiovascular disease, which “improves blood circulation,” based on the TCM theory and practice to the treatment of “blood stasis.” Salvianolate is a water-soluble effective active site of traditional Chinese herb salviae miltiorrhizae, and its main component is magnesium lithospermate B.^[13]^ Magnesium lithospermate B can protect the cardiovascular system via anti-oxidation, anti-inflammation, endothelial protection, myocardial protection, anticoagulation, vasodilatation, anti-atherosclerosis, and reducing the proliferation and migration of vascular smooth muscle cells.^[14]^ It can also reduce the incidence of contrast-induced nephropathy after PCI and is more effective than normal saline.^[15]^ In addition, it has been confirmed that intravenous administration of salvianolate can improve cardiac microcirculatory perfusion and increase cardiac output in rats.^[16]^ Similarly, continuous intravenous salvianolate 10 mg/kg/day for 7 days can significantly improve myocardial microcirculatory reflow in an ischemia-reperfusion minipig model, as well as reduce oxidative stress and myocardial apoptosis.^[17]^ However, there is no evidence to show the effectiveness of Salvianolate injection in the treatment of ISR. Therefore, this study is the first to explore the efficacy of Salvianolate injection in patients with ISR through systematic review and meta-analysis. It aims to provide clinicians with valuable references to complementary therapy and alternative therapy.

## Author contributions

**Conceptualization:** Miao Zhang, Yue Yuan.

**Data curation:** Miao Zhang, Ying Gao.

**Formal analysis:** Miao Zhang, Ruozhu Lu.

**Funding acquisition:** Yue Deng.

**Investigation:** Yue Yuan.

**Methodology:** Miao Zhang, Ying Gao.

**Project administration:** Miao Zhang, Ying Gao.

**Resources:** Miao Zhang, Yue Yuan, Ruozhu Lu.

**Software:** Miao Zhang, Yue Yuan.

**Supervision:** Yue Deng.

**Validation:** Ruozhu Lu, Yue Deng.

**Visualization:** Yue Deng.

**Writing – original draft:** Miao Zhang.

**Writing – review & editing:** Yue Deng.
